# Acidic and alkaline conditions enhance *Staphylococcus aureus* α-toxin pore formation and cytotoxicity

**DOI:** 10.1371/journal.ppat.1014568

**Published:** 2026-09-01

**Authors:** Tristan J. van der Linden, Arnab Chatterjee, Lisette M. Scheepmaker, Debajyoti Chakraborty, Somnath Dutta, Bart W. Bardoel, András N. Spaan

**Affiliations:** 1 Department of Medical Microbiology, University Medical Center Utrecht, Utrecht, The Netherlands; 2 Molecular Biophysics Unit, Indian Institute of Science, Bengaluru, Karnataka, India; Lunds universitet Medicinska fakulteten, SWEDEN

## Abstract

*Staphylococcus aureus* α-toxin (AT) is a potent pore-forming toxin that causes cell damage during infection. The structure and activity of AT have been studied extensively, but the environmental factors that modulate its toxicity remain unclear. Here, we demonstrate that the extracellular pH modulates AT cytotoxicity through two independent mechanisms in human cells. First, we show that acidic conditions enhance AT pore maturation, causing a toxin-intrinsic and ADAM10 independent increase in cytotoxicity after prolonged exposure to AT. Second, we demonstrate that both acidic and alkaline conditions impair the cellular capacity to internalize AT pores, resulting in toxin retention at the cell surface and increased cytotoxicity after pulse intoxication. The cell-intrinsic, pH-dependent sensitization to AT is observed in various human cell types but not for other pore forming toxins. Together, our findings indicate that the extracellular pH modulates AT activity and host cell susceptibility.

## Author summary

*Staphylococcus aureus* is a bacterium that can cause severe invasive infections. *S. aureus* produces many toxins during infection, of which α-toxin (AT) is clinically relevant. AT is a pore-forming toxin that damages host cells by assembling into ring-shaped complexes that insert into the cell membrane to form transmembrane pores. During infection, *S. aureus* may encounter environments with different acidity, such as the skin, inflamed tissues, and the lungs. The impact of the local acidity on AT function is unclear. Here, we show that pH alters AT cytotoxicity through two separate mechanisms. First, acidic conditions enhance AT pore formation, causing an increase in its toxicity. Second, both acidic and alkaline conditions impair a host cell defence pathway that removes AT from the cell membrane. This leads to increased membrane damage after even brief toxin exposure. We observed this pH-dependent sensitization in multiple human cell types, but not with other pore-forming toxins. Together, our findings indicate that local tissue conditions, including the environmental acidity, modulate AT activity and host cell susceptibility.

## Introduction

*Staphylococcus aureus* is a major human pathogen and one of the leading causes of bacterial infections [1]. Most individuals carry *S. aureus* asymptomatically on their skin and in their nostrils, but some people develop infections. Disease manifests with significant clinical variability, ranging from mild infections (e.g., folliculitis, abscesses and cellulitis) to severe infections (e.g., necrotizing pneumonia, and necrotizing skin and soft tissue infections) with poor prognoses [1–5]. The bacterium’s success as a pathogen lies in its ability to adapt to distinct host environments and to deploy a broad arsenal of virulence factors that manipulate and circumvent host defences [6]. Among these, one of the best-characterized is α-toxin (AT, also known as haemolysin-alpha or Hla). AT forms transmembrane pores in host cell membranes and thereby contributes to tissue injury and immune modulation [7–9].

AT is a member of the hemolysin-family of the β-barrel pore forming class toxins (β-PFT) [10]. This group of virulence factors oligomerises on their target cell membrane to form transmembrane pores with membrane-spanning domains that consist of β-barrels [10]. Secreted by *S. aureus* as a monomeric protein, AT binds to its target cell membrane through various membrane lipids and, more efficiently, through the membrane receptor A Disintegrin and Metalloprotease 10 (ADAM10) [7,11,12]. AT subsequently heptamerizes and undergoes various structural changes that promote pore formation [13,14]. The resulting pore permeabilizes the plasma membrane to single ions such as Na^+^, K^+^ and Ca^2+^ [9,15]. The subsequent ion flux triggers the activation of programmed cell death pathways and inflammatory signalling pathways at low toxin concentrations, or direct cell lysis at high concentrations [16–20]. AT pore-formation also induces ADAM10 metalloprotease activity in epithelial cells, leading to cleavage of extracellular matrix components and disruption of epithelial barriers [7,21,22]. Despite the extensive characterization of AT structure and activity, the environmental and host factors that modulate its activity remain incompletely understood.

One environmental factor is the variable pH of host tissues. A neutral pH of ~7.4 is often considered the physiological pH [23,24], but *in vivo* and *ex vivo* studies in animals and humans demonstrate that local pH in tissues is not neutral [25–32]. For instance, pH of healthy stratum corneum ranges from ~4.1 to 6 [24,33–35], while pH of healthy airway surface liquid ranges from pH ~ 7.5-9 [31]. Moreover, infection itself affects the local pH of tissues. The normally neutral dermis is mildly acidic in acute wounds with pH reported of ~6.6 [32,36], and in becomes alkaline in chronic wounds with pH ranging from 7.5-8.9 [32,36–38]. Local pH of bacterial wound infections ranges from 4.5-6.5 [35], and pH in abscesses is mildly acidic with a pH of approximately 6.6 [27,39,40]. Thus, *S. aureus* encounters diverse environmental pH conditions during colonization and infection. Despite the established impact of pH on various biochemical processes, its impact on the virulence potential of *S. aureus* is unknown.

Studies using artificial lipid vesicles, conducted prior to the identification of ADAM10 as the membrane receptor of AT, suggest that AT exhibits enhanced pore formation capacity at acidic pH. This effect has been attributed to a refolding of the stem domain, which forms the membrane-perturbing β-barrel pore. This refolding results in reduced protein stability, increased hydrophobicity of the toxin and enhanced pore formation under acidic conditions [12,41,42]. However, these artificial vesicles consist of a lipid bilayer without surface proteins relevant in AT cytotoxicity, including ADAM10. Additionally, these vesicles have no membrane repair mechanisms [19,43–45]. Thus, it remains unclear whether the observed pH-dependent effects translate to a more complex model target such as nucleated host cells.

Here, we study the effect of the extracellular pH on the cytotoxicity of *S. aureus* AT on human cells. We demonstrate that the extracellular pH modulates AT cytotoxicity through two separate mechanisms. First, acidic conditions enhance AT pore maturation in a toxin-intrinsic manner. Second, both acidic and alkaline conditions cause a cell-intrinsic retention of AT pores at the cell surface. These findings reveal a role of the environmental pH in shaping the host susceptibility to AT.

## Results

### Acidic conditions enhance AT cytotoxicity in an ADAM10-independent manner

**Fig 1:**
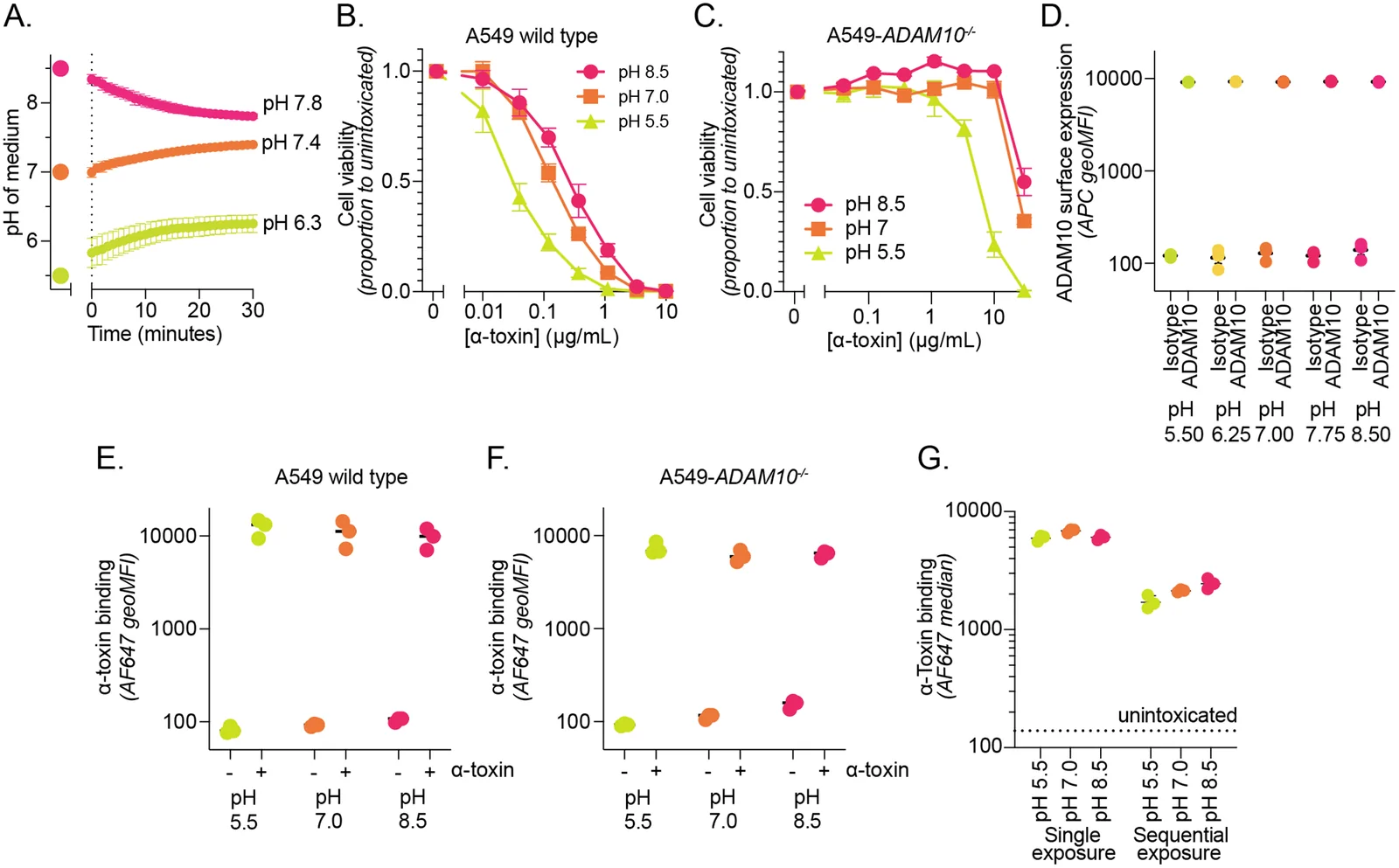
Acidic conditions enhance AT-mediated cytotoxicity but not toxin binding. (A) pH of culture medium over time in cell culture conditions (5% CO_2_, 20% O_2_, 37°C). Each dot represents the mean of a technical triplicate, with *n =* 3 individual experiments. Bars represent mean ± SEM. **(B and C)** Viability after 24 hours treatment with AT of (A) wild-type and **(B)** *ADAM10* knock-out A549 cells. Cells were treated in culture medium calibrated to the indicated pH. Proportion of cell viability is calculated using the unintoxicated control within each pH condition as denominator. Each dot represents the mean cell viability of *n =* 3 individual experiments, each with technical duplicate. Bars represent mean ± SEM. **(D)** Surface expression of the AT receptor ADAM10 on A549 cells after 24 hours of incubation in culture medium calibrated to the indicated pH. After incubation, A549 cells were stained with an anti-ADAM10 antibody, followed by an APC-labelled secondary goat-anti-mouse antibody. Geometric means of ADAM10-APC fluorescent intensity was analysed by means of flow cytometry. **(E)** Binding of Alexa Fluor 647 (AF647)-labelled AT (10 μg/mL) to wild type A549 cells after 10 minutes of exposure at 37°C in culture medium calibrated to the indicated pH. AT-AF647 geometric means were analysed by means of flow cytometry. **(F)** Binding of AT-AF647 (10 μg/mL) to A549-*ADAM10*^-/-^ cells after 10 minutes of exposure at 37°C in culture medium calibrated to the indicated pH. AT-AF647 geometric means were analysed by means of flow cytometry. **(G)** Binding of AF647-labelled AT (1 μg/mL) to A549 cells directly after 30-minute treatment (i.e., single exposure) or after a sequential exposure. In the latter condition, cells were first treated for 30 minutes with Alexa Fluor 488-labelled AT (1 μg/mL), directly followed by a 30-minute treatment with AF647-labelled AT (1 μg/mL). All treatments were performed at 37°C in culture medium calibrated to the indicated pH. AT-AF647 median intensity was analysed by means of flow cytometry. **(D - G)** Each dot represents the mean cell viability of *n* = 3 individual experiments. Bars represent mean ± SEM. Statistical significance was calculated by ordinary one-way analysis of variance (ANOVA) with Tukey’s multiple comparisons test. **(B - G)** Indicated pH represents the pH of the culture media at the start of intoxication with AT.

Given historic data on enhanced pore formation in lipid vesicles in acidic conditions [12,41,42], we first set out to examine the impact of the extracellular pH on AT-mediated cytotoxicity. Alveolar epithelial A549 cells were exposed to AT for 24 hours in culture media pre-calibrated to pH 5.5, 7.0 and 8.5. After 30 minutes under tissue culture conditions, media pH converged to 6.3, 7.4 and 7.8, respectively (Fig 1A, S1A-C Fig). Thus, although we display initial pH values, the actual pH of culture media equilibrated toward neutrality shortly after the start of the experiment. AT was approximately four times more cytotoxic in acidic conditions, as compared with neutral and alkaline pH conditions (Fig 1B, S1 Table). This phenotype is in line with previous work in artificial lipid vesicles and human erythrocytes that lack ADAM10 [12,41,42,46]. We therefore hypothesised that the enhanced cytotoxicity of AT in A549 cells in acidic environment occurs independently of ADAM10. We tested the impact of the extracellular pH on AT-mediated cytotoxicity towards A549 cells in which *ADAM10* was knocked out. As expected, A549-*ADAM10*^-/-^ cells were highly tolerant to AT when compared with wild type cells. However, when these cells were exposed to AT in acidic conditions, cytotoxicity was increased to a similar extent as in the parental cells expressing ADAM10, when compared with neutral and alkaline conditions (Fig 1C, S1 Table). Together, we demonstrate that AT cytotoxicity towards human cells is enhanced in acidic conditions, and that this pH-dependent potentiation does not require ADAM10.

### Extracellular pH does not influence AT binding

Binding of AT correlates with cytotoxicity [46]. To test whether the pH-dependent cytotoxicity of AT is driven by enhanced binding capacity, we first measured ADAM10 surface expression and AT binding on A549 cells. ADAM10 surface expression was similar on A549 cells that were cultured for 24 hours under alkaline, neutral and acidic conditions (Fig 1D). Similarly, AT binding after 10 minutes was not influenced by extracellular pH in A549 cells expressing ADAM10 (Fig 1E). In A549-*ADAM10*^-/-^ cells, even at 10 μg/mL, a concentration at which substantial cytotoxicity is observed in acidic conditions, AT binding was also not significantly affected by pH (Fig 1F). As expected, binding was less efficient in A549-*ADAM10*^-/-^ when compared with wild type cells (S1D-E Fig). Because of the slow kinetics of AT cytotoxicity [11,47], we hypothesised that AT binding continues over time. We tested if continued binding over time occurs in a pH-dependent manner. In an assay in which cells were sequentially exposed to two differentially labelled ATs, we detected additional binding of Alexa Fluor (AF)-647 labelled AT after 30 minutes of pre-exposure to AF488 labelled AT in a pH-independent manner (Fig 1G, Fig S1F). Thus, AT does not saturate the A549 cell surface and continues to bind over time, independently of pH. These data demonstrate that AT binding is not affected by the extracellular pH, suggesting that processes downstream of binding drive the observed pH-dependent cytotoxicity.

### Acidic conditions enhance AT transmembrane pore formation

**Fig 2:**
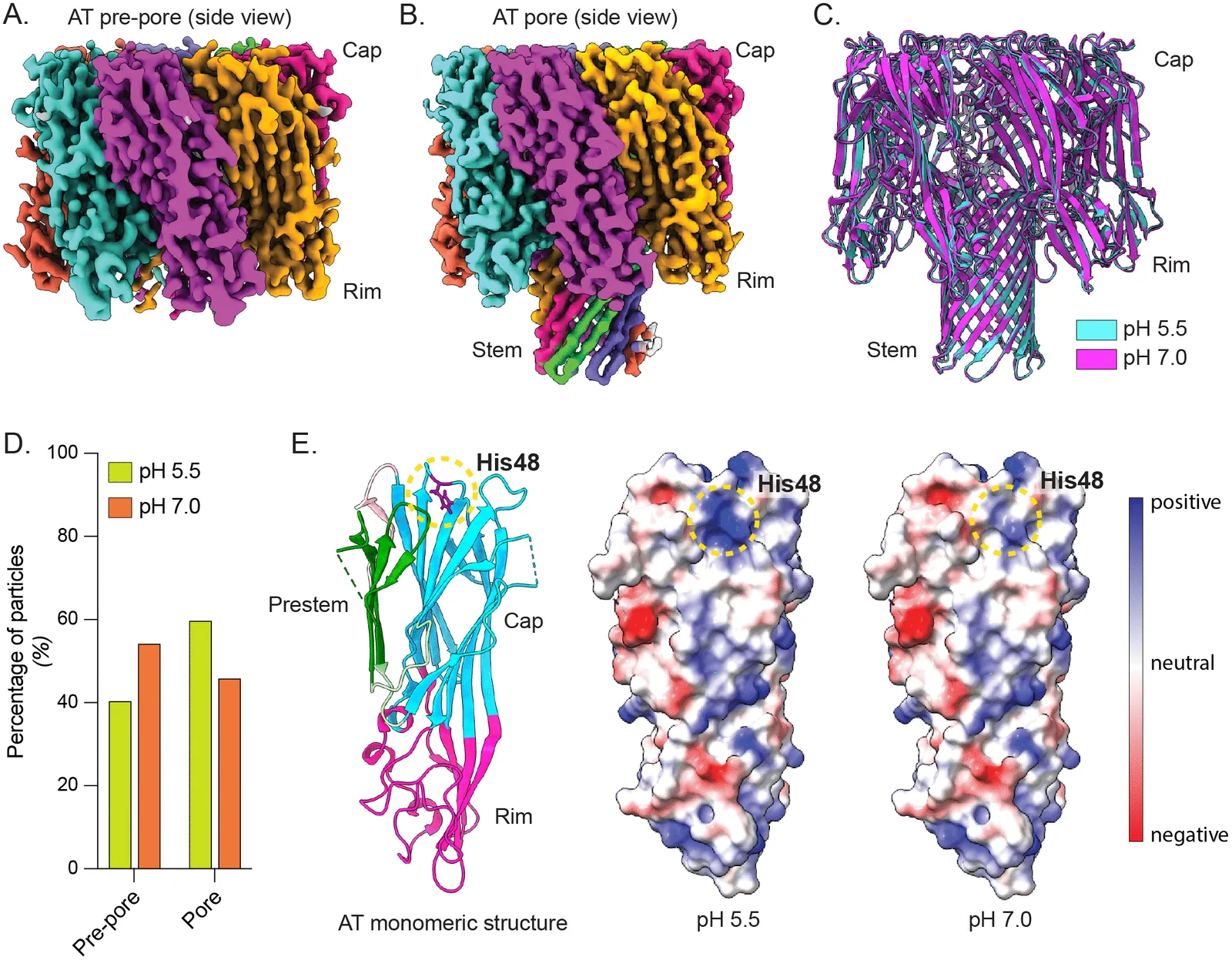
Acidic conditions enhance the AT pre-pore to pore transition. **(A)** Cryo-EM map of the pre-pore state of AT after incubation with A549 cells for 10 minutes at pH 7.0. **(B)** Cryo-EM map of the pore state of AT after incubation with A549 cells for 60 minutes at pH 5.5. **(C)** Alignment of the AT pore structures, after incubation with A549 cells for 60 minutes at pH 5.5 and 7.0. **(D)** Particle distribution pre-pore, and pore states of AT heptamers after incubation with A549 cells for 10 minutes at pH 5.5 or pH 7.0. Indicated pH represents the pH of the culture media at the start of intoxication with AT. **(E)** Left: Positioning of His48 residues in the monomeric crystal structure of the AT_H35A_ monomer (PDB ID: 4YHD). The His48 residue is shown in magenta, amino latch in salmon, the cap domain in blue, pre-stem in green and the rim domain in pink. Middle and right: Electrostatic surface potential analysis of AT_H35A_ monomer (PDB ID: 4YHD) at (middle) pH 5.5 and (right) pH 7.0 (blue: positive charge, white: neutral charge, red: negative charge). The yellow dashed circle marks the positioning of the His48 residue.

We aimed to establish whether pH-dependent changes in the conformation of the AT heptamer may underlie the enhanced cytotoxicity observed in acidic conditions. We performed single-particle cryogenic electron microscopy (cryo-EM) analyses to characterize the particle distribution of the oligomeric AT after 10- and 60-minute incubation periods with A549 cells under acidic and neutral pH. After an incubation period of 10 minutes, we observed both partially assembled oligomeric intermediates and complete circular heptamers in both pH conditions (Fig S2). 3D classification of the heptameric structures revealed populations of pre-pores and transmembrane pores (S3 and S4 Figs). Further refinement of the 3D reconstructions yielded high-resolution pore and pre-pore structures (Figs 2A and 2B, S5A-B Fig). Structural alignment revealed no significant differences of the pores at pH 5.5 and 7.0 (root mean square deviation (RMSD) = 0.575 Å, Fig 2C). However, after both 10- and 60-minutes incubation periods, we detected a higher proportion of transmembrane pores than pre-pores in acidic conditions, but not in neutral conditions (Fig 2D, Fig S5C). These data suggest that acidic conditions enhance the AT pre-pore to pore transition, causing the cell surface to be covered by more fully formed AT pores in acidic conditions than in neutral conditions. To understand the molecular basis of this enhanced pore formation, we performed *in silico* modelling of the surface charge of the monomeric toxin (PDB ID: 4YHD) in pH 5.5 and 7.0 [13]. The His48 residue in the cap domain was predicted to be protonated at acidic pH, but not neutral pH, while other residues in the pre-stem domain or in the junction of the pre-stem and the cap domain remained unchanged in acidic conditions (Fig 2E). Given proximity of His48 to the pre-stem, its charge alteration may facilitate pre-stem release at low pH [13,48]. We aimed to test this hypothesis using a AT_H48K_ mutant, as lysine displays a + 1 charge across the tested pH range, equivalent to histidine at low pH [13]. This substitution abolished hemolytic activity in all pH conditions (Fig S5D). This is in line with a previous study where His48 substitution either abolished or reduced AT hemolytic activity [49]. Thus, His48 amino acid substitution itself does not seem to be tolerated, precluding definitive testing of the mechanism. Combined, these data indicate that acidic conditions enhance AT transmembrane pore formation, pointing towards a structural basis for the enhanced cytotoxicity in acidic conditions.

### Acidic and alkaline conditions enhance AT-mediated membrane permeabilization

**Fig 3:**
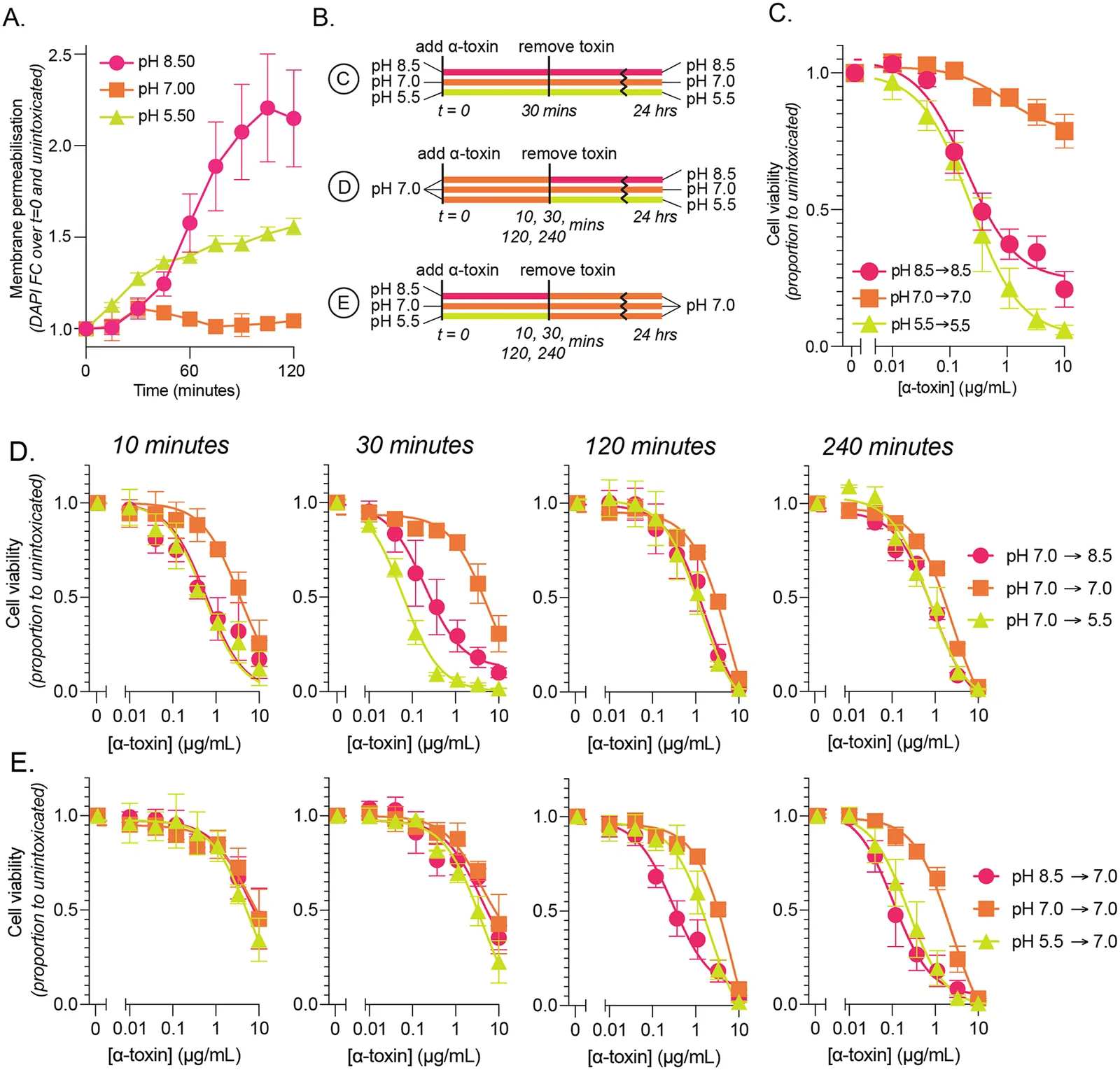
Cells are resistant to short exposures of AT in neutral pH, but are sensitized by acidic and alkaline conditions within a time window of 30 and 240 minutes of intoxication. **(A)** AT (1 μg/mL)-mediated permeabilization of A549 cells in culture medium calibrated to the indicated pH. Permeabilization was quantified through DAPI fluorescent intensity and is displayed as fold-change (FC) over values at t = 0 minutes and unintoxicated control of respective pH condition. Indicated pH represents the pH of the culture media at the start of exposure to AT. **(B)** Treatment conditions in C-E. **(C)** Viability of A549 cells after AT exposure of 30 minutes, followed by removal of unbound toxin and incubation in toxin-free culture media to a total duration of 24 hours. Each dot represents the mean cell viability of *n* = 3 individual experiments, each with technical duplicate. Bars represent mean ± SEM. **(D and E)** Viability of A549 cells after AT exposure of a duration as indicated above D, followed by removal of unbound toxin and incubation in toxin-free culture media to a total duration of 24 hours. **(D)** Cells were exposed to AT in neutral pH, and further incubated in altered pH media. **(E)** Cells were exposed to AT in altered pH, and further incubated in neutral pH. Each dot represents the mean cell viability of *n =* 3 individual experiments, each with technical duplicate. Bars represent mean ± SEM. **(C-E)** Indicated pH represents the calibrated pH of the culture media at the start of each experimental step. Proportion of cell viability is calculated using the unintoxicated control within each pH condition as denominator. Each dot represents the mean cell viability of *n =* 3 individual experiments, each with technical duplicate. Bars represent mean ± SEM.

The loss in membrane integrity that causes membrane permeabilization to molecules larger than single ions is a secondary event that occurs as a downstream consequence of AT pore formation [50–54]. Considering the enhanced capacity to form mature pores in acidic conditions, we investigated the potential of AT to permeabilize the membrane of cells in various extracellular pH conditions. We tested AT-mediated membrane permeabilization on A549 cells using a membrane-impermeant dye that becomes fluorescent upon binding to DNA. 1 µg/mL of AT caused permeabilization within two hours under acidic and alkaline conditions, but not in neutral conditions (Fig 3A, Fig S6A). Thus, whereas cytotoxicity after 24 hours of continuous exposure was enhanced exclusively in acidic conditions, both acidic and alkaline conditions enhanced AT-mediated membrane permeabilization within two hours. Given the relatively short duration of exposure that is required for AT to mediate membrane permeabilization in non-neutral pH, we examined the effect of a pulse exposure to AT. We exposed A549 cells to AT for 30 minutes in culture media with a calibrated pH and subsequently incubated the cells overnight in toxin-free media without changing media pH (Fig 3B). In line with our observations studying membrane permeabilization, cells treated in neutral pH displayed almost no loss in viability, while cells treated in acidic and alkaline pH were highly susceptible to AT cytotoxicity (Fig 3C, S1 Table). Interestingly, continued exposure to AT in acidic conditions caused even more cytotoxicity than pulse exposure in acidic conditions, but not in alkaline conditions (Fig S6B, S1 Table). Taken together, and in contrast to cells continuously exposed to AT, A549 cells are tolerant to a short AT pulse in neutral pH but are susceptible to cell death in both acidic and alkaline conditions.

### Acidic and alkaline pH sensitizes cells to AT within 30 and 240 minutes of intoxication

For human nucleated cells, the interaction between AT and the target cell consists of four discrete phases. The first two phases, binding and pore formation, are toxin-intrinsic [11,41,47]. The following phases, consisting of a loss in membrane integrity (membrane permeabilization) and subsequent caspase-mediated cell death, are cell-intrinsic and require multiple hours to complete [8,9,19,50,51,55,56]. To study the role of the extracellular pH on the cell-intrinsic phases, we performed a series of pulse exposure cytotoxicity assays in which we exposed cells for defined durations at either neutral or altered pH and subsequently exchanged the media from neutral pH to altered pH, or vice versa (Fig 3B). Exposure to AT for 10 and 30 minutes at neutral pH with subsequent overnight incubation at altered pH resulted in a markedly enhanced cytotoxicity in both alkaline and acidic media (Fig 3D, S1 Table). In contrast, exposure to AT for 10 and 30 minutes at altered pH followed by overnight incubation at neutral pH resulted in minimal cytotoxicity with no effect of pH (Fig 3E, S1 Table). Interestingly, pH-dependent potentiation of AT cytotoxicity was strongly reduced when cells were exposed to AT for 120 or 240 minutes at neutral pH followed by overnight incubation at non-neutral pH (Fig 3D, S1 Table). Exposure of cells to AT for the same durations but in opposite pH conditions revealed a potentiation of cytotoxicity in alkaline (after 120 and 240 minutes) and acidic (after 240 minutes) conditions (Fig 3E, S1 Table). Thus, AT cytotoxicity is enhanced by the extracellular pH between 30 and 240 minutes after exposure in A549 cells. A549-*ADAM10*^*-/-*^ cells did not display a significant loss in cell viability after a pulse exposure of AT, irrespective of the pH (Fig S6C). Combined, these data suggest that the sensitization to AT pulses in non-neutral pH is caused by modulation of cellular processes occurring shortly after AT pore formation.

### pH-dependent potentiation of AT occurs in various cell types, but is not a universal feature of β-PFTs

**Fig 4:**
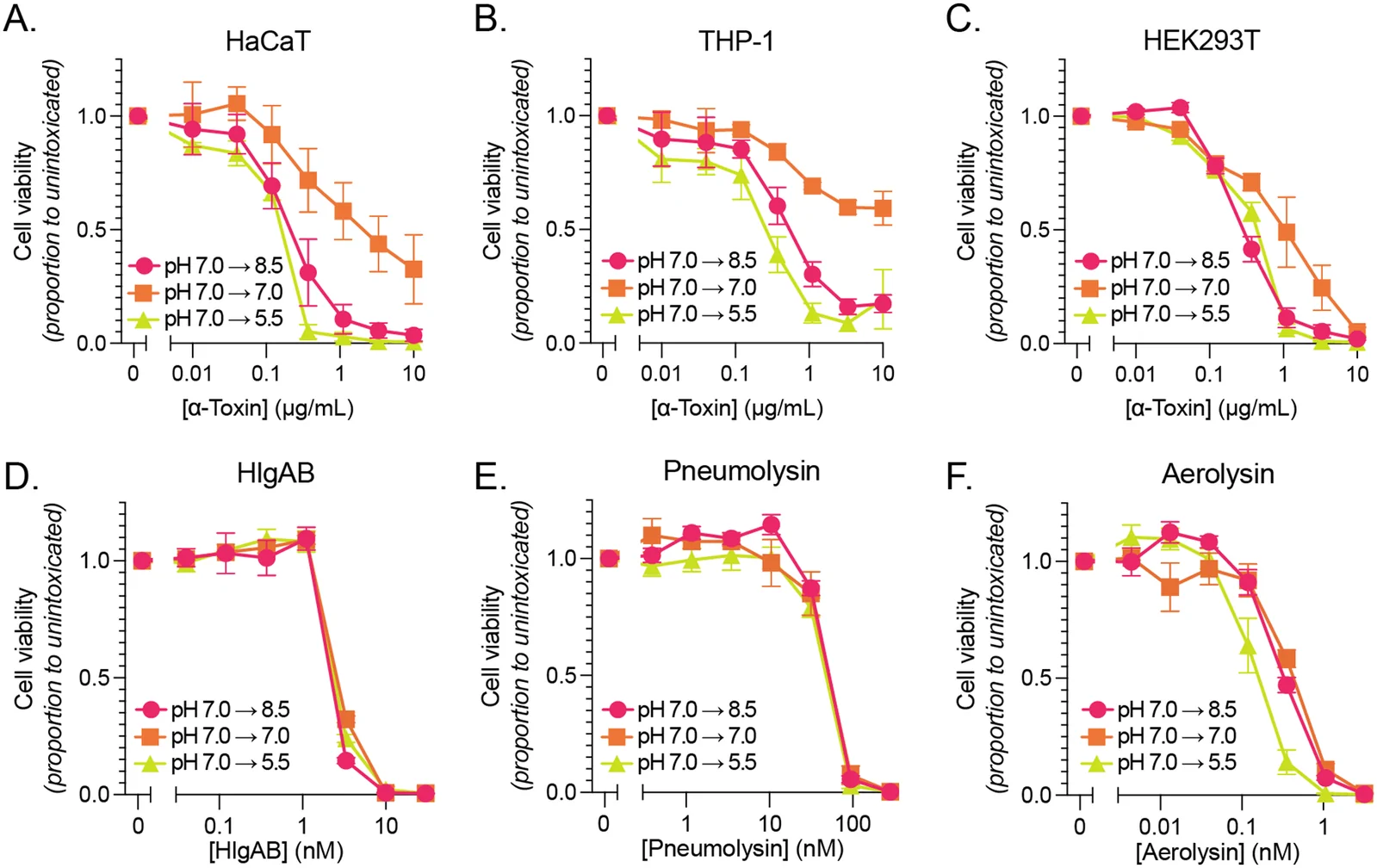
pH-mediated potentiation of AT cytotoxicity is not cell type-specific, but is not a universal feature of β-PFTs. (A - C) Viability of (A) HaCaT, (B) THP-1 and (C) HEK293T cells after AT exposure of 30 minutes in neutral pH culture medium, followed by removal of unbound toxin and incubation to a total duration of 24 hours in toxin-free culture media that has been calibrated to the indicated pH. (D - F) Viability of A549 cells after (D) HlgAB, (E) pneumolysin and (F) aerolysin exposure of 30 minutes in neutral pH culture medium, followed by removal of unbound toxin and incubation to a total duration of 24 hours in toxin-free culture media that has been calibrated to the indicated pH. (D) A549 cells were made permissive to HlgAB cytotoxicity through overexpression of CXCR1. (A - F) Proportion of cell viability is calculated using the unintoxicated control within each pH condition as denominator. Each dot represents the mean cell viability of *n =* 3 individual experiments, each with technical duplicates. Bars represent mean ± SEM. Indicated pH represents the pH of the culture media at the start of intoxication.

To examine if pH-dependent sensitivity to pulse exposures of AT is an A549 cell line-specific phenotype, we also tested other cell types. We intoxicated the HaCaT human keratinocytic cell line, HEK293T epithelial cells, and the THP-1 monocytic cell line with AT for 30 minutes in neutral pH media, after which we removed the unbound fraction of AT and replaced the culture media with either acidified or alkalized pH culture media for overnight incubation. The cells tested displayed variations in their sensitivity to pulse exposures of AT under neutral pH. However, all displayed a significantly enhanced sensitivity to AT elicited cytotoxicity at both acidic and alkaline conditions (Figs 4A-C, S1 Table). Thus, cells of various origin display a pH-dependent sensitivity to pulse exposures of AT. Next, we set out to test whether other β-PFTs display a similar phenotype of pH-dependent cytotoxicity [10]. We tested the staphylococcal bicomponent leukocidin HlgAB (like AT belonging to the family of hemolysins, forming octameric pores), the aeromonas toxin aerolysin (belonging to the family of aerolysins, forming heptameric pores), and the streptococcal pneumolysin (belonging to the family of cholesterol-dependent cytolysins, forming large pores with up to 44 subunits) using the same experimental design for pulse exposures in A549 cells. Thus, this panel includes β-PFTs with (i.e., HlgAB and aerolysin) or without (i.e., pneumolysin) dependency on a proteinaceous membrane receptors, and with varying pore sizes [10,57–59]. HlgAB and pneumolysin did not display any pH-dependency in their cytotoxicity (Figs 4D and 4E, S1 Table). Aerolysin was slightly more cytotoxic exclusively in acidic conditions (Fig 4F, S1 Table). In total, A549 cells did not exhibit a similar pH-dependent sensitivity to short exposures of these toxins as for AT. We therefore conclude that the pH-dependent cytotoxicity of pulse exposures is not a universal feature of β-PFTs. Combined, these data suggest that toxin-specific characteristics of AT - but not HlgAB, pneumolysin or aerolysin - facilitate a pH-dependent sensitization across the cell types tested.

### A549 cells internalize AT following pore formation

**Fig 5:**
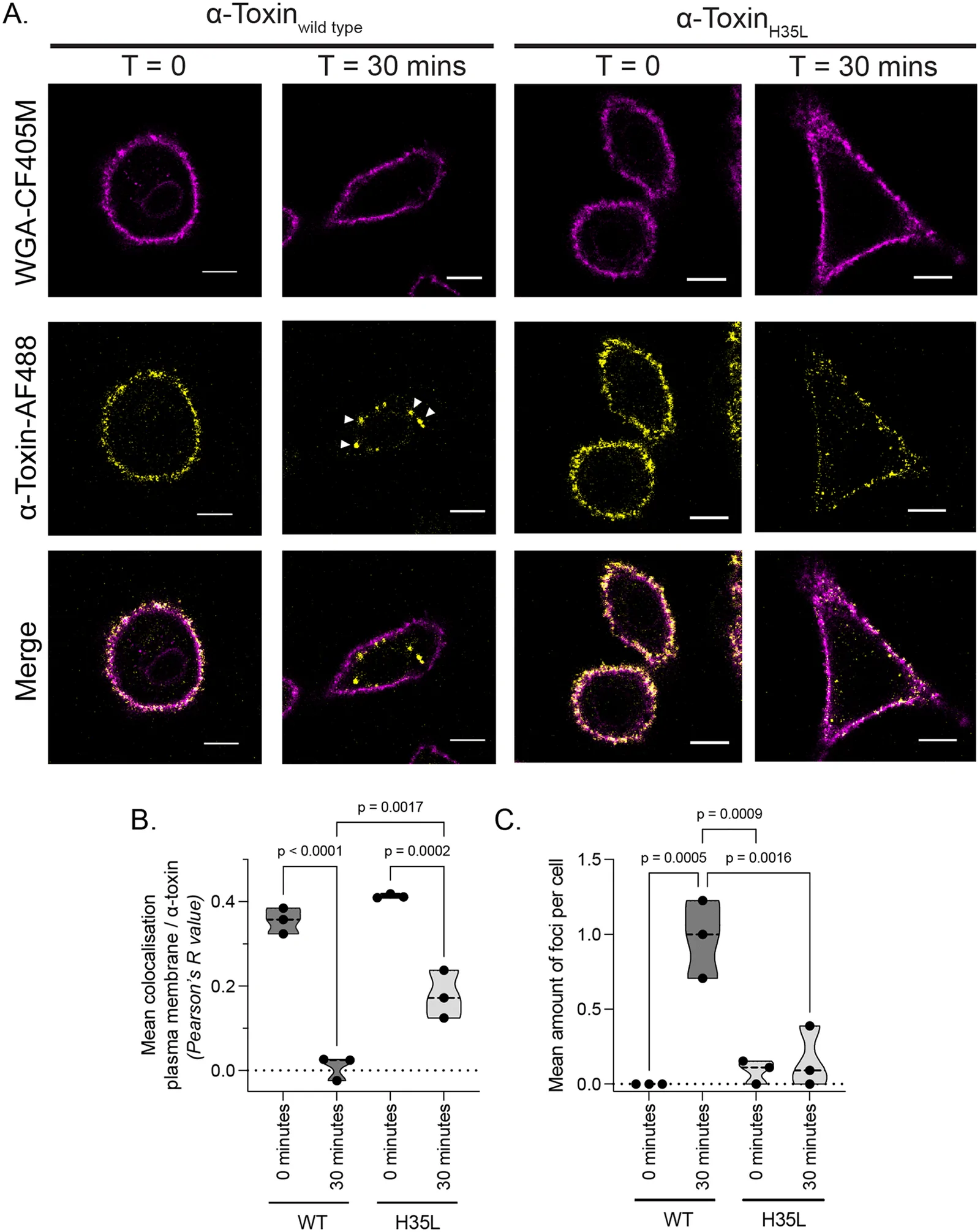
A549 cells internalize AT as a result of pore formation. **(A)** Representative confocal images of A549 cells treated with Alexa Fluor 488-labelled AT_wild-type_ or AT_H35L_ (both 1 μg/mL) for 10 minutes at 37°C. Cells were subsequently placed on ice (indicated as T = 0) or incubated for 30 minutes at 37°C (Indicated as T = 30) prior to staining with CF405M-labelled Wheat Germ Agglutinin (WGA) and fixation. White arrows indicate examples of AT foci larger than 0.75 μm^2^, which were counted in the quantitative analysis. Scale bars are 10 μm. **(B)** Colocalization (Pearson’s correlation) of the WGA-stained membranes and AT in A549 cells. Each point represents the mean colocalization in cells of n ≥ 12 images per individual experiment, with 3 individual experiments. Statistical significance was calculated by ordinary one-way analysis of variance (ANOVA) with Tukey’s multiple comparisons test. **(C)** Mean amount of AT foci per cell with a size greater or equal to 0.75 μm^2^. Each point represents the mean colocalization in cells of n ≥ 12 images per individual experiment, with 3 individual experiments. Statistical significance was calculated by ordinary one-way ANOVA with Tukey’s multiple comparisons test.

AT-mediated membrane permeabilization is triggered by pore formation at the cellular surface [7,9,45,60]. Following pore formation, AT is internalized and sequestered into large multivesicular bodies. This process enhances the cellular resilience to AT [19,45,61]. We hypothesized that acidic and alkaline extracellular environments may impact the cellular capacity to internalize AT pores, and tested AT localization over time by means of confocal microscopy. A549 cells were exposed to AT, after which the cells were placed in toxin-free, neutral pH culture media. After 30 minutes of continued incubation, AT displayed no colocalization with the plasma membrane (Figs 5A and 5B). Additionally, large intracellular AT foci were formed (Figs 5A and 5C). To verify whether this internalization of AT is dependent on pore formation, we subsequently used the AT_H35L_ single amino acid exchange mutant, which can bind to ADAM10 but is incapable of forming transmembrane pores [7,62]. After 30 minutes of incubation in toxin-free media, AT_H35L_ displayed a reduction, but no loss of colocalization with the plasma membrane (Figs 5A and 5B). Further, no intracellular foci were formed, as was the case with wild type AT (Figs 5A and 5C). Together, these data confirm that A549 cells internalize the toxin at neutral pH, and that this process requires pore formation.

### AT is retained at the plasma membrane at non-physiological pHs

**Fig 6:**
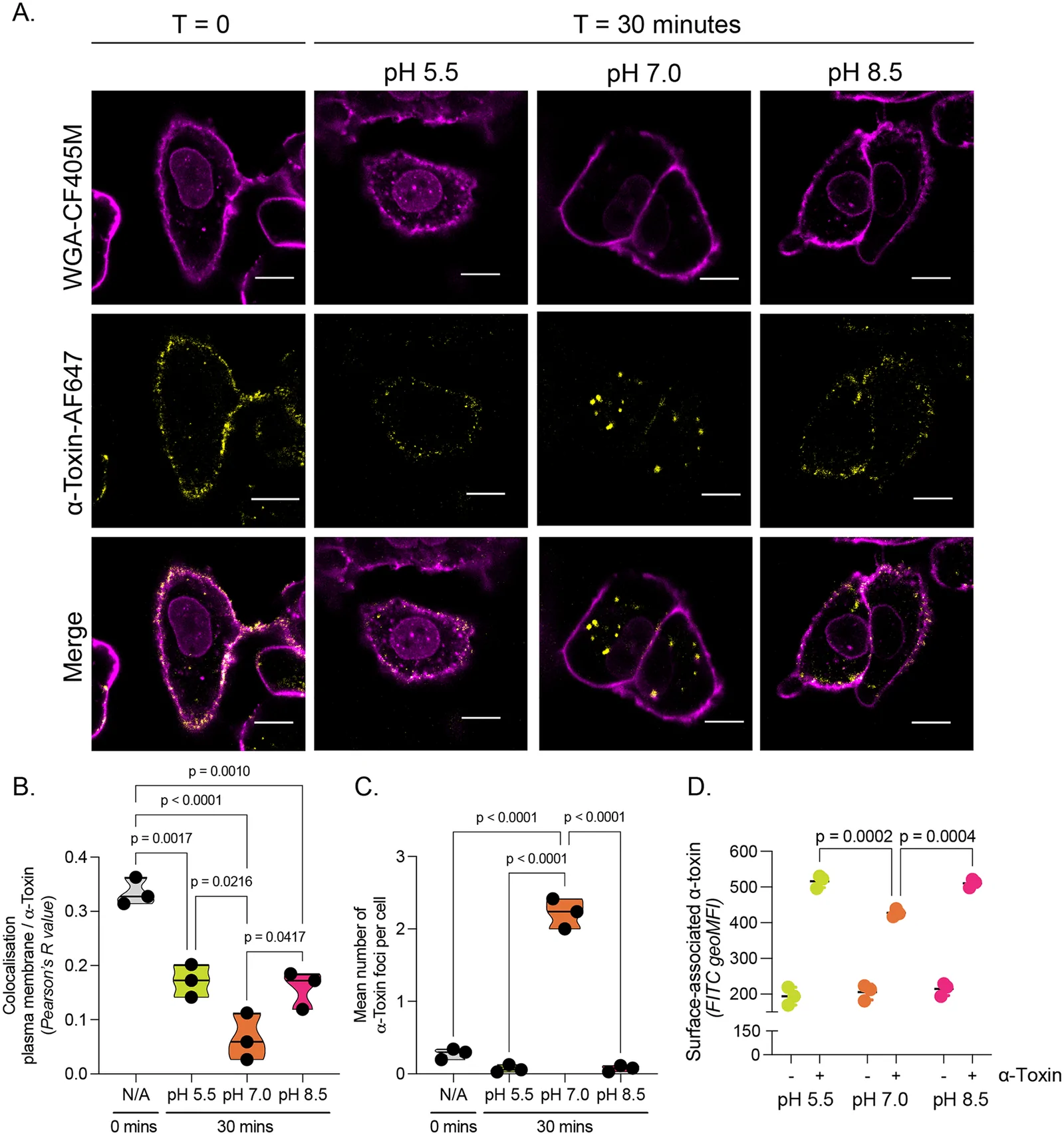
AT is retained at the plasma membrane in acidic and alkaline conditions. **(A)** Representative confocal images of A549 cells treated with Alexa Fluor 647-labelled AT (1 μg/mL) for 10 minutes at 37°C in neutral pH culture medium. Cells were subsequently placed on ice (indicated as T = 0) or incubated for 30 minutes at 37°C (Indicated as T = 30) in culture medium calibrated to the indicated pH prior to staining with CF405M-labelled Wheat Germ Agglutinin (WGA) and fixation. Scale bars are 10 μm. **(B)** Colocalization (Pearson’s correlation) of the WGA-stained membranes and AT in A549 cells. Each point represents the mean colocalization in cells of *n ≥* 12 images per individual experiment, with 3 individual experiments. Statistical significance was calculated by ordinary one-way analysis of variance (ANOVA) with Tukey’s multiple comparisons test. **(C)** Mean number of AT foci per cell with a size greater than or equal to 0.75 μm^2^. Each point represents the mean colocalization in cells of *n ≥* 12 images per individual experiment, with 3 individual experiments. Statistical significance was calculated by ordinary one-way ANOVA with Tukey’s multiple comparisons test. **(D)** Surface associated AT on A549 cells treated with 1 μg/mL C-terminal his-tagged toxin for 10 minutes at 37°C in neutral pH culture medium, and subsequently incubated for 30 minutes at 37°C in culture medium calibrated to the indicated pH. After incubation, A549 cells were stained with an anti-his-tag antibody, followed by a FITC-labelled secondary goat-anti-rabbit antibody. Geometric means of AT-FITC fluorescent intensity was analysed by means of flow cytometry. Statistical significance was calculated by ordinary one-way ANOVA with Tukey’s multiple comparisons test. **(A – D)** Indicated pH represents the pH of the culture media in which cells were incubated after removal of unbound AT.

Since AT-mediated membrane permeabilization is dependent on the extracellular pH, we subsequently tested if the internalization of AT is affected by the extracellular pH. We exposed A549 cells to AT for 10 minutes, after which the cells were placed in toxin-free, altered pH culture media. After 30 minutes of continued incubation, AT remained relatively more colocalized with the plasma membrane in acidic and alkaline conditions, when compared with neutral pH conditions (Figs 6A and 6B). Additionally, only negligible amounts of intracellular AT foci were formed in cells intoxicated in acidic and alkaline media (Figs 6A and 6C). These data indicate that AT remains at the cell surface at acidic and alkaline conditions, as compared with neutral conditions. To validate these findings, we exposed cells to the same toxin treatment and subsequently stained the histidine-tag of the plasma membrane-associated fraction of AT. Through flow cytometry, we detected more plasma membrane-associated AT in cells intoxicated under acidic or alkaline pH conditions, when compared with neutral pH condition (Fig 6D). Combined, these data suggest that retention of AT at the cell surface under acidic and alkaline conditions underlies the observed increase in permeabilization and cytotoxicity at non-neutral pH conditions.

## Discussion

**Fig 7:**
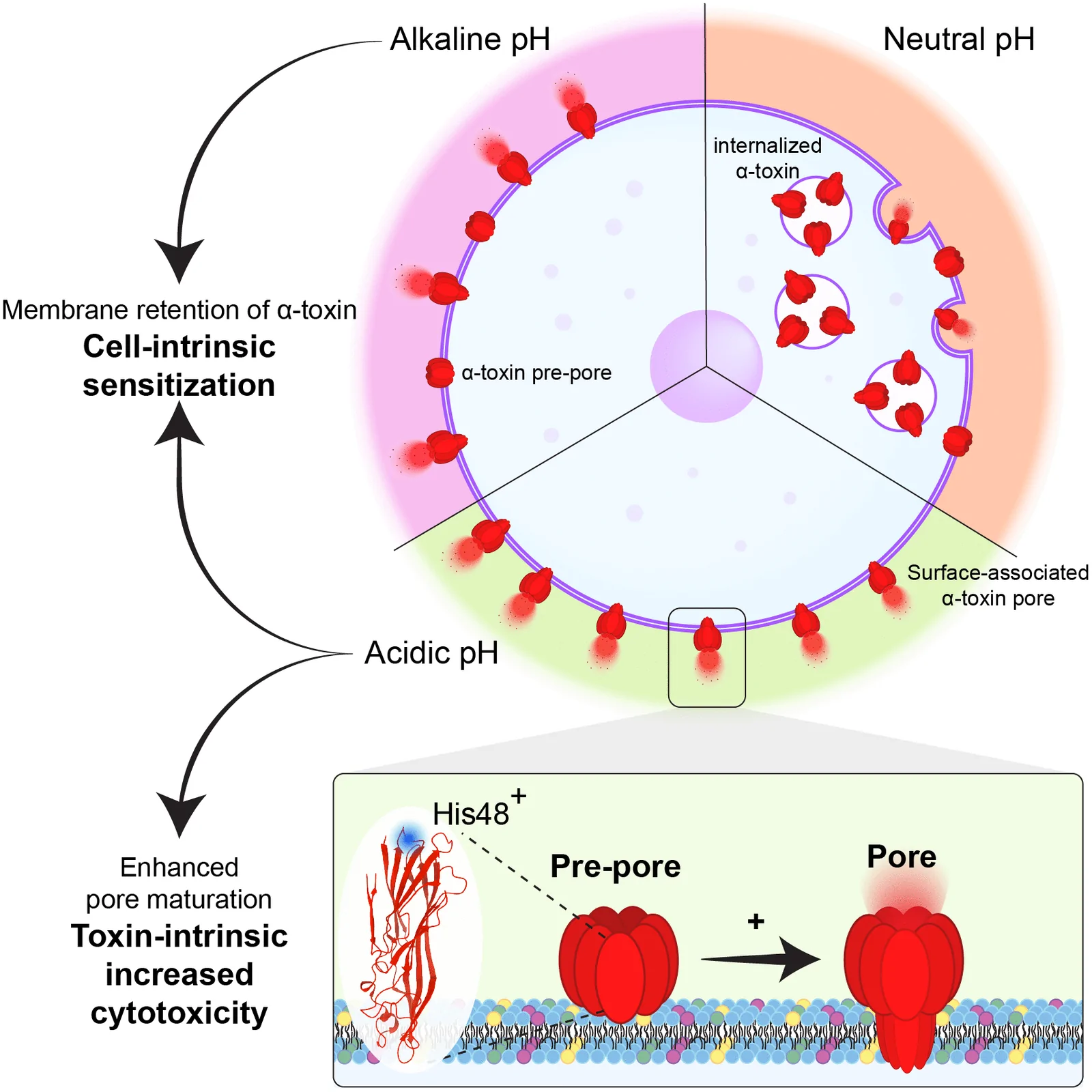
Model of mechanism of pH-dependent cytotoxicity of AT. Extracellular pH modulates AT cytotoxicity through two independent mechanisms. Acidic conditions enhance AT pore-maturation, causing a toxin-intrinsic increase in cytotoxicity. Both acidic and alkaline conditions cause a retention of AT on the cell surface, resulting in heightened cell sensitivity. AT crystal structure was acquired from the RCSB Protein Data Bank, with accession number 4YHD. Figure was made using Adobe Illustrator.

We demonstrate that the extracellular pH modulates AT cytotoxicity through two independent mechanisms in cultured human cells (Fig 7). First, using single particle cryo-EM, we show an increase in AT pore maturation in acidic conditions. The increased pore-formation causes enhanced cytotoxicity after prolonged exposure, independently of ADAM10. This phenotype resembles data obtained in artificial lipid vesicles and human erythrocytes that lack ADAM10 [12,41,46]. Second, we show a cell-intrinsic sensitization to AT cytotoxicity in non-neutral conditions due to a diminished capacity to internalize AT, resulting in the retention of AT at the cell surface. Prior reports indicate that the internalization of AT is critical to the cellular defence against AT-mediated membrane damage [19,45,63,64]. By impairing AT-internalization, non-neutral pH conditions cause the sensitization of cells that are otherwise resistant to AT pulse exposures. Combined, non-neutral pH conditions enhance AT cytotoxicity due to both a toxin-intrinsic increase of pore maturation and a cell-intrinsic defect of immunity to AT.

Our finding that AT displays a toxin-intrinsic, pH-dependent pore-forming capacity is consistent with biochemical studies of AT from the 1980s and 1990s [12,41,42]. After prolonged exposures, AT displayed enhanced cytotoxicity exclusively in acidic conditions. The increased cytotoxicity was also seen in cells lacking ADAM10, and was previously reported in artificial lipid vesicles and human erythrocytes [12,41,46]. ADAM10 is essential for AT-induced barrier disruption during infection [7,21,22]. ADAM10 facilitates AT binding, but the receptor is not strictly required for cytotoxicity as the toxin can also directly interact with various membrane lipids [7,65]. Given the increased pore-formation by AT at acidic pH in artificial lipid vesicles [12,41,42], and its enhanced cytotoxicity under acidic conditions in cells independently of ADAM10 expression, our findings suggest that the augmented pore maturation under acidic conditions occurs irrespective of ADAM10. Our finding that pH did not affect AT binding suggests that enhancement is downstream of toxin binding to the target membrane. Through *in silico* modelling in monomeric AT, we predict a protonation of His48 in acidic conditions, with no additional charge alterations in the N-terminal region or at the cap and pre-stem interface. His48 locates close to the pre-stem segment and is crucial in toxin function, with mutations at this site reducing lytic activity without altering the binding to target cells [48,49]. Prior work in lipid vesicles proposed that acidic conditions increase flexibility and refolding of the stem domain, leading to enhanced pore formation [41,42,66]. We therefore hypothesize that protonation of His48 destabilizes the cap-pre-stem interaction and promotes pre-stem release, contributing to pre-pore to pore transition in a pH-dependent manner.

Cells internalize AT pores under neutral pH conditions, thereby preventing membrane permeabilization and cytotoxicity [19,45]. However, AT internalization is inhibited under acidic and alkaline conditions, leading to the retention of AT at the cell surface. The resulting accumulation of AT pores at the cell surface causes increased cell permeability and, ultimately, cell death. The impact of ADAM10 on the pH-dependent resilience to AT pulses remains to be established, because cells were resistant to AT pulse exposure in the absence of ADAM10, regardless of pH-condition. Prior work indicates that AT internalization is a cell-type dependent mechanism of intrinsic immunity, where, e.g., HEK293 cells had more retention of AT at the plasma membrane [45]. This is in line with our findings that not all cell types displayed equal susceptibility to AT pulses at neutral pH, and that pH-mediated increases in pulse cytotoxicity were relatively minor in HEK293T cells. AT pulse cytotoxicity was modulated by the extracellular pH between 30 minutes and four hours after addition of the toxin, a timeframe during which internalization can meaningfully limit membrane permeabilization [45]. Combined, the cell-intrinsic immunity to AT under neutral pH conditions is driven by a cell-type dependent capacity to internalize the toxin. This resilience can be circumvented through alterations in the extracellular pH.

The outcome of AT cytotoxicity is partially determined by the duration of AT exposure. Under neutral pH, cells display a relative tolerance to pulses of AT, but not to prolonged exposures to the toxin. The sensitivity to sustained intoxication may be explained through our finding that AT continues to accumulate on target cells over time. This continuous binding of AT potentially enables AT to overcome cell-intrinsic immunity against AT and ultimately induce cytotoxicity. Alkaline conditions inhibited cell-intrinsic immunity against AT, causing the toxin to inflict most of its pH-mediated additional cytotoxicity very soon after start of intoxication, but prolonged exposures did not result in even further enhanced cytotoxicity. The cell-intrinsic immunity against AT also failed in acidic conditions, resulting in the sensitivity to pulse exposures of AT. However, AT proceeded to cause even further cytotoxicity after prolonged intoxications in acidic conditions. This suggests that, in acidic conditions, both the inhibition of cell-intrinsic immunity to AT and the toxin-intrinsic increase in pore formation contribute additively to cytotoxicity. Interestingly, other β-PFTs did not display a pH-dependent cellular resilience to pulse exposures. At physiological concentrations, AT causes cell permeabilization and cell death in a span of hours, whereas HlgAB and pneumolysin cause cell death after only minutes [52,67–70]. The mechanisms that drive cell-intrinsic immunity to AT may act in a toxin-specific fashion, providing a potential explanation for the relatively slow-acting nature of AT. Together, our findings highlight a crucial role of cell-intrinsic immunity to AT in determining the kinetics of cytotoxicity.

*In* and *ex vivo* studies in animals and humans indicate that *S. aureus* encounters environments with diverse pH during colonization and infection [24,27,31–33,37,39,40,71]. In the experimental conditions in this work, the pH of media at the start of experiments (pH 5.5-8.5) rapidly converges to relatively mildly acidic to mildly alkaline (pH 6.3-7.8). Thus, even small deviations from neutral pH can profoundly affect AT pore-formation and cell-intrinsic immunity to AT. Beyond its extracellular lifestyle, *S. aureus* also resides intracellularly, where the phagolysosome represents a particularly relevant compartment [72–74]. This organelle is actively acidified [75–77] to facilitate acid-activated host enzymes in killing and digesting internalized bacteria [78–80]. Work in professional phagocytes shows that *S. aureus* can escape the phagolysosome to kill the host cell [74,81]. Phagolysosomal escape of *S. aureus* is largely driven by expression of the genes encoding Leukocidin AB (LukAB or LukGH) and phenol-soluble modulins (PSMs) [82–85], but a potential contribution of AT at acidic pH in conjuction with other virulence factors remains to be established. AT promotes survival of phagocytosed *S. aureus* by preventing acidification of the phagosome in human macrophages [86], but it remains to be established if AT pore formation contributes to intracellular survival and phagolysosomal escape. Combined, future studies are needed to assess the impact of local pH on *S. aureus* virulence potential and tissue tropism *in vivo*.

While our study focuses on pH-dependent modulation of AT activity after secretion, environmental pH also serves as an input for two regulatory systems of *S. aureus* virulence. First, the quorum-sensing accessory gene regulon (*agr*) is repressed *in vitro* in acidic (pH ~ 5.3 and ~6.5) and alkaline (pH ~ 8.0) growth medium, with corresponding reduction in *hla* transcription [28,87–89]. Second, acidic pH (pH 5.5) suppresses the SaeRS two-component system *in vitro*, dampening AT expression [28,90]. In contrast, *ex vivo* expression studies in *S. aureus* grown on intact murine skin revealed expression of *agr* and *sae*, despite the acidic nature of this tissue [91]. This suggests that currently underappreciated regulatory networks and environmental factors may compensate to maintain toxin production in non-neutral pH. One candidate is the GraRS two-component system, which senses acidic pH and induces envelope remodelling and antimicrobial resistance in *S. aureus* [92]. *graRS* deletion also suppresses *agr* and *hla* expression, indicating crosstalk between GraRS and the *Agr*/*Hla* axis under at least some conditions [93,94]. Further work is needed to explore how local tissue pH, in concert with other environmental signals, shapes AT production *in vivo*.

Recent studies have identified the impact of human genetic and immunological determinants in individual patients on their susceptibility to severe *S. aureus* infections [60,95]. Human OTULIN haploinsufficiency underlies life-threatening staphylococcal disease by disrupting the cell-intrinsic immunity to AT in nonleukocytic cells [60]. Patients suffer from episodes of life-threatening necrosis of their skin and/or lungs [60,95,96]. Upon binding of AT to ADAM10, the receptor is retained at the surface of these patients’ cells. ADAM10 colocalizes with AT after internalization, suggesting that AT is retained on the surface of the patients’ cells, thereby enhancing AT cytotoxicity [7]. It remains to be established if the susceptibility of patients with OTULIN haploinsufficiency to AT cytotoxicity is further modulated by the extracellular pH. AT-neutralizing antibodies and compounds have shown promising results in preclinical studies [97–103], but results of clinical trials focusing on prevention and treatment of pneumonia have disappointed [104,105]. Antibody-antigen interactions are often pH sensitive [106], suggesting that the therapeutic efficacy of AT-neutralizing antibodies may be negatively impacted by the environmental pH. Beyond the role of the environmental pH in staphylococcal disease, our findings highlight the importance of well-defined conditions to experimental reproducibility. Indeed, we have observed substantial consequences in the cellular behaviour with even minor variations in culture medium pH. Thus, we recommend control of culture medium pH through the use buffers such as HEPES or calibrating the culture medium to a set pH, in addition to maintaining a CO_2_-rich atmosphere.

In conclusion, we demonstrate that the extracellular pH modulates AT cytotoxicity by enhancing pore maturation in acidic conditions and by reducing the cell-intrinsic immunity to AT under acidic and alkaline conditions. Thus, we identify the extracellular pH as a determinant of AT cytotoxicity.

## Methods

### Cell culture

For the cryo-EM experiments, A549 cells were maintained in Dulbecco′s Modified Eagle′s Medium (DMEM-Sigma-Aldrich), supplemented with 20% foetal bovine serum, and 1% antibiotic in a 37°C and 5% CO_2_ incubator. For all other experiments, A549 (RRID:CVCL_A549, not authenticated), HaCaT (RRID:CVCL_0038, not authenticated), HEK293T (RRID:CVCL_0063, not authenticated) and THP-1 cells (RRID:CVCL_0006, not authenticated) were cultured in Dulbecco’s modified Eagle’s medium (DMEM), supplemented with GlutaMAX (Gibco), 10% heat-inactivated foetal bovine serum (HI-FBS) (Gibco) and 25 mM HEPES (Gibco). All cells were regularly tested for mycoplasma contamination using the MycoAlert Mycoplasma Detection Kit (Lonza Bioscience) and used in experiments exclusively in case no contamination was detected. All cells were maintained at 37°C with 5% CO_2_.

### Calibration of culture medium pH

For each experiment, DMEM supplemented with GlutaMAX (Gibco), 1% HI-FBS (Gibco) and 25 mM HEPES (Gibco) was calibrated to a set pH with HCl or NaOH. Using a method adapted from Michl et al. (S1A and S1B Figs) [107], the pH-dependent changes of absorbance of phenol red at 435 nm and 560 nm in 60 µL of culture media were measured every minute for 30 minutes. Measurements were performed in a 96-well plate in the CLARIOstar Plus plate reader (BMG LabTech) with the atmospheric control unit (BMG LabTech) at cell culture conditions of 37°C with 5% CO_2_.

### Generation of A549-ADAM10^-/-^ cells

A549 cells were mixed with the gene knock-out kit V2 for human ADAM10 (Synthego) - which contains a pool of three synthetic guide RNA targeting ADAM10 – in the presence of TrueCut Cas9 protein V2 (Thermo Fisher Scientific) according to manufacturers’ protocols. Cell transfection was subsequently performed through electroporation with the Neon Transfection system (Thermo Fisher Scientific) using the cell line-specific settings provided by the manufacturer (i.e., pulse voltage 1200V, pulse width 30 ms, 2 pulses). After 7 days, clonal populations were generated by limited dilution cloning, expanded, and validated through sequencing and detection of surface-expressed ADAM10 through by means of flow cytometry. For validation through sequencing, genomic DNA (gDNA) was isolated from 3 × 10^5^ cells using the Wizard Genomic DNA purification kit (Promega). Exon 1 of *ADAM10* was amplified by nested polymerase chain reaction (PCR), purified with the GeneJet PCR purification kit (Thermo Fisher Scientific) according to manufacturer’s protocol and subsequently Sanger sequenced. For validation through flow cytometry, 5 × 10^4^ cells were incubated for 30 minutes in a total volume of 50 µL on ice with 3 µg/mL mouse anti-human ADAM10 antibody (clone 11G2, Novus), washed three times and subsequently incubated with fluorescein isothiocyanate (FITC)-conjugated goat-anti-mouse antibody (1:50, Dako) in DMEM supplemented with GlutaMAX (Gibco), 1% HI-FBS (Gibco) and 25 mM HEPES (Gibco). FITC was subsequently detected on the BD FACSverse (BD Biosciences) and analysed using FlowJo (version 10, BD Life Sciences).

### Generation of A549-CXCR1^+^ cells

To make A549 cells permissive to HlgAB intoxication, a lentiviral expression system was used to induce stable expression of HlgAB’s membrane receptor, the human CXCR1. The complete *CXCR1* cDNA was cloned in the BIC-PGK-PuroR;EF1A dual promotor lentiviral vector, derived from Weijer, M. L. et al. [108]. This lentiviral vector uses the human EF1A promotor to facilitate potent expression, and expresses the selection marker PuroR. Virus was produced in 24-well plates using standard lentiviral production protocols and the third-generation packaging vectors pMD2G-VSVg, pRSV-REV, and pMDL/RRE. Briefly, in a total volume of 1 mL, 0.25 µg lentiviral vector and 0.25 µg packaging vectors were cotransfected in HEK293T cells by using 1.5 ul Mirus LT1 transfection reagent (Sopachem). After 72 hours, 100 µl unconcentrated supernatant containing lentivirus was used to infect 5 × 10^4^ A549 cells. 72 hours after addition of virus, selection of transduced cells was performed using 2 µg/mL puromycin (Thermo Fisher Scientific), after which cells were expanded to confluency. Clonal populations were generated by means of Fluorescent-Activated Cell Sorting (FACS). 1 × 10^5^ cells were incubated in a total volume of 50 µL on ice with 3 µg/mL mouse anti-human CXCR1 antibody (clone 42705, R&D Systems), followed by FITC-conjugated goat-anti-mouse antibody (1:50, Dako). Using the MA900 Multi-Application Cell Sorted (Sony), single cells with high CXCR1 surface expression were sorted into separate wells of a 96-well plate. Cells were subsequently expanded in DMEM, supplemented with GlutaMAX (Gibco), 10% HI-FBS (Gibco), 25 mM HEPES (Gibco) and penicillin-streptomycin until confluent.

### Production and fluorescent labelling of recombinant toxins

For the cryo-EM experiments, the purification process of the recombinant AT was carried out following the previously described protocol [11]. The AT used for all other experiments and pneumolysin were produced using standard procedures [109]. In brief, the AT gene of *Staphylococcus aureus* strain Newman and the *ply* gene of *Streptococcus pneumoniae* were synthesized and modified by addition of a C-terminal cysteine, followed by a thrombin cleavage site and a His-tag. These were cloned into the pET28A vector (Novagen). The AT_H35L_ and AT_H48K_ mutants were generated using the QuickChange II XL Site-Directed Mutagenesis kit (Agilent) following vendor protocol on the same vector. The resulting vectors were transformed into *Escherichia coli* BL21(DE3) (Thermo Fisher Scientific). For a typical sample preparation, bacteria were grown in Luria Broth at 37 °C until A600 of 0.5, prior to addition of 1 mM isopropyl β-d-thiogalactopyranoside to induce protein expression. After 4 hours, cells were harvested by spinning at 4 °C for 15 minutes at 3500 × *g*. Cells were subsequently lysed under denaturing conditions, which consistently yielded higher protein recovery compared to native lysis. In brief, the supernatants were discarded, and pellets were directly lysed through resuspension in a denaturing lysis buffer (50 mM Tris, 500 mM NaCl, 6 M Guanidine hydrochloride, pH 8) and sonification. His-tagged toxins were isolated using 5 mL HisTrap HP (Cytiva) columns according to manufacturer’s protocol. The eluted proteins were subsequently dialysed into PBS and cleared of aggregated protein by means of centrifugation followed by filtration through a 0.22 µm membrane filter before size exclusion chromatography. To isolate monomeric AT, the protein was loaded into the Superdex 75 Increase 10/300 GL columns (Cytiva) according to manufacturer’s protocol, with PBS as running buffer. The isolated protein was concentrated using Amicon Ultra-4 centrifugal filter unit with 10 kDa molecular cutoff (Merck). The SEC peak fraction for recombinant monomeric AT was either stored at 4°C until use, or immediately fluorescently labelled. To label the purified AT, it was incubated with 10 × molar excess of either Alexa Fluor 647-C2-Maleimide (Thermo Fisher Scientific) or Alexa Fluor 488-C5-Maleimide (Thermo Fisher Scientific) at room temperature overnight. The reaction was quenched with 0.01 M glutathione. Excess dye and glutathione were removed through SEC, and concentrated as described above. Purity was analysed on SDS-PAGE, showing a single monomeric band for AT. HlgAB and aerolysin were produced and purified as previously described [60,68].

### Cryo-EM sample preparation

Before the toxin treatment, around 1 × 10^6^ A549 cells per well were taken and seeded in a 6-well plate. The pH of the cultured cells was adjusted to indicated pH in FBS-free media before intoxication. AT was immediately added to wells at a 1mg/ml concentration and incubated for either 10 minutes or 1 hour in a 37°C, 5% CO2 incubator. After toxin treatment, the cells were harvested by centrifugation at 1000 rpm. 3µl of the harvested A549 cells incubated with toxins was applied onto the freshly glow-discharged R1.2/1.3 300 mesh copper grids (Quantifoil; Electron Microscopy Sciences). Samples were immediately blotted for 6 s with a positive blot force of 5 using an FEI Vitrobot Mark IV. Following blotting, grids were rapidly vitrified by plunge-freezing into liquid ethane.

### Cryo-EM data collection

Cryo-EM data were collected using a 200 kV Talos Arctica transmission electron microscope (Thermo Scientific) equipped with a K2 Summit Direct Electron Detector (Gatan Inc.). Movies were acquired automatically using Latitude-S [110] (Digital Micrograph - GMS 3.5) with a total dose of 40 e-/Å2, with an exposure time of 8 seconds distributed for 20 frames at a magnification of 54,000x at the effective pixel size of 0.92 Å.

### Cryo-EM single-particle data processing

Single-Particle Analysis (SPA) was carried out in the cryoSPARC 4.5 [111]. Drift and gain corrections for the recorded movies were performed using Patch Motion Correction, followed by estimation of contrast transfer function (CTF) parameters using Patch CTF Estimation. Micrographs were then filtered through a curated exposure selection with a resolution cutoff of 8 Å, and those with a resolution poorer than 8 Å were excluded from subsequent processing. Previously deposited heptameric cryo-EM map (EMD-62307) was used to create a template, and the templates were used for template-picker-based picking. The particles were extracted with a box size of 280 pixels (pixel size of 0.92 Å). Several rounds of reference-free 2D classification were performed to remove incorrectly picked or poorly aligned particles and to separate arc-like intermediate species from fully assembled heptameric particles. The particles used for 3D reconstruction (lipid membrane-toxin clusters) were highlighted in yellow lines in the cryo-EM micrographs (Fig S4). The ab initio models were reconstructed from the best heptameric 2D class averages to use as references for heterogeneous refinement using C7 symmetry. The high-resolution 3D classes having a decent amount of particle number were subjected to non-uniform refinement using C7 symmetry, which includes the estimation of spherical aberration, anisotropic magnification, per-particle defocus, and CTF refinement. The global resolution was estimated using the Fourier shell correlation (FSC) criterion at the 0.143 threshold [112].

### Model building and structure refinement

The cryo-EM structure of heptameric AT (PDB ID: 9 KG3) was rigid-body fitted into the corresponding cryo-EM maps using UCSF ChimeraX [113] and subsequently used as the initial template for atomic model building using Coot [114] and PHENIX [115] of both the pore and pre-pore conformations. The map to model validation was carried out using PHENIX (Fig S5A).

### Analysis and visualization of cryo-EM maps and atomic models

The cryo-EM maps and atomic models were validated using Phenix, and EMringer [116], Molprobity [117], respectively. Local resolution of the pore and pre-pore maps was performed using Cryosparc, and the results were visualized in ChimeraX [113] (Fig S5B). The detailed structural analysis was performed using ChimeraX [113]. Maps were sharpened using DeepEMhancer for visualization [118]. Electrostatic calculations of the AT monomer (PDB ID: 4YHD) were performed using the Adaptive Poisson-Boltzmann Solver on the PDB2PQR server [13,119].

### Cell viability assays

A549, HaCaT, THP-1 and HEK293T cells were seeded in a 96-well plate, with 1 × 10^4^ cells per well. For the HEK293T cells, the plate was coated with 0.01% poly-L-lysin (Sigma-Aldrich). Cells were synchronised for 24 hours in DMEM supplemented with GlutaMAX, 1% FBS and 25 mM HEPES, with pH 7.0, at 37°C and 5% CO_2_. Cells were subsequently intoxicated with toxin in dose-response in culture medium with indicated pH, at 37°C and 5% CO_2_. At shown timepoints, culture medium containing toxin was aspirated and toxin-free medium with indicated pH was added. Cells were then incubated at 37°C and 5% CO_2_. 24 hours after the addition of toxin, cell viability was assessed by measuring intracellular ATP contents with the CellTiter-Glo Luminescent Cell viability Assay (Promega) according to manufacturer’s protocol. Luminescence (emission: 555–70) was measured on the CLARIOstar Plus plate reader (BMG LabTech), with gain set at 3600 (arbitrary unit) and focal height of 10.6 mm. Luminescence values of each sample were divided by the raw luminescence value of the unintoxicated controls within each respective pH-condition to transform the raw data into proportion of unintoxicated.

### Hemolysis assay with recombinant AT

Sheep erythrocytes (Analytichem) were washed thrice in PBS that had been set to pH 7.0, before resuspending in PBS that was adjusted to the indicated pH to a final concentration of 2 × 10^8^ cells/mL. After cells were exposed to recombinant AT or 1% triton X-100 at a final concentration of 1.33 × 10^8^ cells/mL in a total reaction volume of 150 μL for 30 minutes at 37°C. Samples were centrifuged for 5 minutes at 500 × *g*, and 100 μL of cell-free lysates were used to measure absorbance (OD405 nm) in the CLARIOstar Plus plate reader (BMG LabTech). Absorbance values of unintoxicated controls within each respective pH-condition were subtracted to control for background lysis, after which the hemolysis was calculated as a percentage of the absorbance values of triton X-100 exposed samples.

### Membrane permeabilization assay

A549 cells were seeded in a 96-well plate, with 1 × 10^5^ cells per well. Cells were synchronised for 24 hours in DMEM supplemented with GlutaMAX, 1% FBS and 25 mM HEPES, which had been set to pH 7.0, at 37°C and 5% CO_2_. After, cells were exposed to 1 µg/mL recombinant AT in DMEM supplemented with GlutaMAX, 1% FBS, 25 mM HEPES and 2.5 µg/mL 4′,6-diamidino-2-phenylindole (DAPI, Thermo Fisher Scientific), with indicated pH. DAPI (excitation: 360–20, emission: 460–30) was measured every 15 minutes for 120 minutes in the CLARIOstar Plus plate reader (BMG LabTech) with gain set at 1300 (arbitrary unit) and focal height of 2 mm, with the atmospheric control unit (BMG LabTech) at cell culture conditions of 37°C with 5% CO_2_.

### ADAM10 surface expression assay

3 × 10^5^ A549 cells were cultured for 24 hours in a 6-well plate in DMEM supplemented with GlutaMAX, 1% FBS, 25 mM HEPES with indicated pH, at 37°C and 5% CO_2_. After, cells were trypsinized and incubated in a total volume of 50 µL on ice with 5 µg/mL APC-conjugated mouse anti-human ADAM10 antibody (clone SHM14, Biolegend) for 30 minutes. Cells were washed three times, fixed with 2% formaldehyde and APC was subsequently detected on the BD FACSverse (BD Biosciences) and analysed using FlowJo (version 10, BD Life Sciences).

### AT binding, saturation and membrane retention assays

5 × 10^4^ A549 cells were trypsinized and placed in a total reaction volume of 50 µL, in DMEM supplemented with GlutaMAX, 1% FBS, 25 mM HEPES calibrated to indicated pH. For the AT binding assay, cells were immediately incubated for 10 minutes with AT directly labelled with Alexa fluor 647 in dose-response at 37°C and 5% CO_2_. Cells were washed three times, fixed with 2% formaldehyde and Alexa fluor 647 was subsequently detected on the BD FACSverse (BD Biosciences) and analysed using FlowJo (version 10, BD Life Sciences). For the membrane saturation assays, cells were immediately incubated for 30 minutes with 1 µg/mL AT directly labelled with Alexa fluor 488 in DMEM supplemented with GlutaMAX, 1% FBS, 25 mM HEPES with indicated pH, at 37°C and 5% CO_2_. Culture medium containing toxin was then aspirated, and cells were subsequently incubated for 30 minutes with 1 µg/mL AT directly labelled with Alexa fluor 647 in culture medium with indicated pH at 37°C and 5% CO_2_. Cells were washed three times, fixed with 2% formaldehyde and Alexa fluor 488 and Alexa Fluor 647 were subsequently detected on the BD FACSverse (BD Biosciences) and analysed using FlowJo (version 10, BD Life Sciences). For the membrane retention assays, cells were immediately incubated for 10 minutes with 1 µg/mL AT directly labelled with Alexa fluor 647 in DMEM supplemented with GlutaMAX, 1% FBS, 25 mM HEPES with pH 7.0, at 37°C and 5% CO_2_. Culture medium containing toxin was then aspirated, and cells were subsequently incubated for 30 minutes in toxin-free culture medium with indicated pH. Cells were then washed three times and incubated on ice with rabbit anti-His-tag antibody (clone D3I1O, 1:400, Cell signaling Technology), followed by FITC-conjugated goat-anti-rabbit antibody (1:40, Sigma-Aldrich). Cells were washed three times, fixed with 2% formaldehyde and FITC was subsequently detected on the BD FACSverse (BD Biosciences) and analysed using FlowJo (version 10, BD Life Sciences).

### AT localization assay

A549 cells were seeded on 8-well µ-slide (Ibidi), and synchronised for 24 hours in DMEM supplemented with GlutaMAX, 1% FBS and 25 mM HEPES, which had been set to a pH of 7.0, at 37°C and 5% CO_2_. Cells were incubated for 10 minutes with 1 µg/mL AT labelled with either Alexa Fluor 488 or Alexa Fluor 647 in DMEM supplemented with GlutaMAX, 1% FBS, 25 mM HEPES calibrated to pH 7.0, at 37°C and 5% CO_2_. Subsequently, cells were either fixed with 2% formaldehyde or further incubated for 30 minutes in toxin-free culture medium with indicated pH and then fixed with 2% formaldehyde. Cells were then incubated with 3 µg/mL Wheat Germ Agglutinin conjugated CF405M fluorophore (Biotium) in PBS for 5 minutes on ice to label cell membranes. Afterwards, cells were imaged using 63 × oil objective on a Leica SP5 confocal microscope (Leica Microsystems). Confocal images were analysed using Fiji and Cellprofiler [120,121]. By segmenting cell boundaries to identify AT-positive cells, AT colocalization with the cell membrane and the number of AT foci larger than 0.75 µm^2^ were attributed to parental cells.

### Statistical analyses

The data were analysed using one-way analysis of variance (ANOVA) with Tukey post hoc correction for multiple comparisons. All statistical analyses were performed using Prism V10. The significance level was set as *P ≤ 0.05*.

## Supporting information

S1 Fig Binding of AT to A549 cells is not affected by pH. (A) Absorbance spectrum of phenol red (PhR) in DMEM supplemented with Glutamax, 1% foetal bovine serum and 25 mM HEPES, and calibrated to indicated pH through titration with HCl or NaOH. Arrows indicate the wavelengths (435 nm and 560 nm) used to determine culture medium pH. (B) pH-dependence of the 560/435 nm ratio, with fitted curve used for the interpolation of culture medium pH in C. Each dot represents the mean 560/435 nm absorbance ratio of *n =* 3 individual experiments, each with technical triplicates. (C) Cellular ATP levels in A549 cell incubated for 24 hours in cell culture conditions, in culture media calibrated to the indicated pH, as a proxy for metabolic rate. Each dot represents the mean of a technical duplicate, with *n =* 3 individual experiments. Bars represent mean ± SEM. Statistical significance was calculated by ordinary one-way analysis of variance (ANOVA) with Tukey’s multiple comparisons test. (D and E) Binding of Alexa Fluor 647 (AF647)-labelled AT to (left) A549 wild type or (right) A549-ADAM10^-/-^ cells after 10 minutes of exposure at 37°C in culture medium calibrated to the indicated pH. AT-AF647 geometric means were analysed by means of flow cytometry. Dots represent mean of *n =* 3 individual experiments. Bars indicate mean ± SEM. (F) Binding of AF488-labelled AT (1 μg/mL) to A549 cells prior to a sequential exposure to AF647-labelled AT. Cells were treated with AF488-labelled AT for 30 minutes at 37°C in culture medium calibrated to the indicated pH. AT-AF488 median intensity was analysed by means of flow cytometry. Each dot represents the average AF488 median of *n =* 3 individual experiments. Bars represent mean ± SEM.
(TIF)

S2 Fig Reference-free 2D class averages of arc-like intermediate species of AT. (A) 2D class averages of AT intermediate states after incubation with A549 cells for 10 minutes at pH 5.5. (B) 2D class averages of AT intermediate states after incubation with A549 cells for 10 minutes at pH 7.0.
(TIF)

S3 Fig Cryo-EM data processing pipelines of the AT incubated with A549 cells. (A) Cryo-EM data processing pipeline of the AT incubated with A549 cells for 10 minutes at pH 7.0. (B) Cryo-EM data processing pipeline of the AT incubated with A549 cells for 10 minutes at pH 5.5. (C) Cryo-EM data processing pipeline of the AT incubated with A549 cells for 60 minutes at pH 7.0. (D) Cryo-EM data processing pipeline of the AT incubated with A549 cells for 60 minutes at pH 5.5.
(ZIP)

S4 Fig Cryo-EM analysis of AT after incubation with A549 cells. (A) Top and side views of the pre-pore state of AT after incubation with A549 cells for 10 minutes at pH 7.0. The density corresponding to the N-terminal is highlighted using a yellow arrow. (B) Map-to-model fitting of pre-pore state. (C) Atomic model of the pre-pore state of AT. The upper transmembrane segment (TMs) is highlighted. (D) Cryo-EM map of the pore state of AT after incubation with A549 cells for 60 minutes at pH 5.5. (E) Map-to-Model fitting of pore state.
(TIF)

S5 Fig Acidic conditions enhance the AT pre-pore to pore transition. (A) Map-to-model validation of the pore state of AT after incubation with A549 cells for 60 minutes at pH 5.5 and the pre-pore of AT after incubation with A549 cells for 10 minutes at pH 7.0. (B) Local resolution estimation of pore state of AT after incubation with A549 cells for 60 minutes at pH 5.5 and pre-pore state of AT after incubation with A549 cells for 10 minutes at pH 7.0. (C) Particle distribution pre-pore, and pore states of AT heptamers after incubation with A549 cells for 60 minutes at pH 5.5 or pH 7.0. (D) Susceptibility of sheep erythrocytes to (left) AT_wild type_ or (right) AT_H48K_. Percentage of hemolysis is calculated using the unintoxicated control within each pH condition as denominator, and cells treated with 1% triton X-100 as positive control with complete lysis. Each dot represents the mean cell viability of *n =* 1 individual experiment with technical duplicate.
(TIF)

S6 Fig Acidic and alkaline conditions sensitize A549 cells to AT pulses. (A and B) Permeabilization of A549 cells in culture medium calibrated to the indicated pH, containing (A) no toxin or (B) 1 μg/mL AT. Permeabilization was quantified through DAPI fluorescent intensity. (C) Viability of A549 cells after either prolonged, 24-hour exposure to AT, or 30-minute pulse exposure to AT with further incubation in toxin-free culture media to a total duration of 24 hours. Indicated pH represents the pH of cell culture medium at the start of the intoxication and, after removal of toxin, in case of pulse exposures. Each dot represents the mean cell viability of *n =* 3 individual experiments, each with technical duplicate. Bars represent mean ± SEM. (D) Viability of *ADAM10* knock-out A549 cells after AT exposure of 30 minutes, followed by removal of unbound toxin and incubation in toxin-free culture media to a total duration of 24 hours. Indicated pH represents the pH of cell culture medium during and after exposure to AT, respectively. Each dot represents the mean cell viability of *n =* 3 individual experiments, each with technical duplicate. Bars represent mean ± SEM.
(TIF)

S1 Table Half maximal effective concentrations (EC50) of AT. *: Statistical significance was calculated by ordinary one-way ANOVA with Tukey’s multiple comparisons test. **: Statistical significance was calculated by unpaired t test with Welch’s correction.
(XLSX)

S2 Table Cryo-EM Data collection, image processing, and refinement for AT structures in the presence of A549 cells at different pH. (XLSX)

S3 Table Cryo-EM map and model validation for AT structures in the presence of A549 cells at different pH. (XLSX)

## Abbreviations

β-PFT: β-barrel pore-forming toxin
ADAM10: A Disintegrin and Metalloprotease 10
AF: Alexa Fluor
AT: α-toxin
Cryo-EM: Cryogenic electron microscopy
CTF: contrast transfer function
CXCR1: C-X-C motif chemokine receptor 1
DAPI: 4′,6-diamidino-2-phenylindole
DMEM: Dulbecco’s modified Eagle’s medium
EC_50_: Half-maximum effective concentration
FCS: Fourier shell correlation
HI-FBS: Heat-Inactivated Foetal bovine Serum
HlgAB: γ-hemolysin AB
OTULIN: OTU deubiquitinase with linear ubiquitin activity
RMSD: Root mean square deviation
SPA: Single-Particle Analysis
WT: Wild type
